# Impact of heparin‐aspirin therapy in patients with recurrent pregnancy loss characterized by thrombophilia resistant to low‐dose aspirin therapy: A retrospective study

**DOI:** 10.1002/rmb2.12643

**Published:** 2025-03-26

**Authors:** Tomoko Ichikawa, Takami Watanabe, Yumene Kubota, Shigeru Matsuda, Daisuke Shigemi, Sayuri Kasano, Ryoko Yokote, Mirei Yonezawa, Nozomi Ouchi, Yasuyuki Negishi, Yoshimitsu Kuwabara, Toshiyuki Takeshita, Shunji Suzuki

**Affiliations:** ^1^ Department of Obstetrics and Gynecology Nippon Medical School Tokyo Japan; ^2^ Department of Microbiology and Immunology Nippon Medical School Tokyo Japan; ^3^ Takeshita Ladies Clinic Shinjuku‐ku Japan

**Keywords:** aspirin, heparin, lupus anticoagulant (LAC), recurrent pregnancy loss, thrombophilia predispositions

## Abstract

**Purpose:**

Heparin and aspirin (HA) therapy is used for antiphospholipid syndrome (APS)‐associated recurrent pregnancy loss (RPL). Low‐dose aspirin (LDA) is recommended for thrombophilic predispositions, such as antiphospholipid antibodies that do not completely meet the Sydney classification criteria for APS, protein S deficiency, factor XII deficiency and increased platelet aggregation. However, no established strategy exists for cases where LDA is ineffective. Therefore, this study aimed to identify the characteristics of RPL cases unresponsive to LDA and to determine whether HA is more effective than LDA in such cases.

**Methods:**

A total of 913 LDA‐treated pregnancies were categorized into live births and miscarriages, and their characteristics were retrospectively analyzed.

**Results:**

The live birth rates following one, two, or three or more LDA therapies were 78.5%, 61.5% and 16.7%, respectively. Live birth rates were significantly lower when lupus anticoagulant (LAC) aPTT was positive but did not qualify as obstetric APS following LDA therapy (birth rates: 33.3%, *p* = 0.048). Three or more LDA therapies decreased the live birth rate, whereas HA therapy significantly increased the live birth rate (*p* = 0.0019).

**Conclusions:**

HA therapy is recommended over repeated LDA treatment, particularly when LAC aPTT is positive but does not qualify as obstetric APS.

## Graphical abstract

Anticoagulation is used for thrombophilic predisposition with recurrent pregnancy loss, but when aspirin therapy fails to achieve live birth, repeated aspirin therapy is not effective, and heparin therapy is effective, especially when LAC aPTT is positive, which is evident.

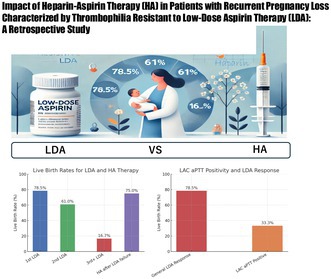

## 1 INTRODUCTION

Recurrent pregnancy loss (RPL) is a condition characterized by two or more miscarriages or stillbirths, affecting approximately 5% of reproductive‐age women.^1^ It is caused by various factors, including uterine morphology abnormalities and parental chromosomal abnormalities, among which thrombophilic predisposition is considered a contributing factor.^2^, ^3^

Thrombophilic predisposition can be either acquired or hereditary. Acquired antiphospholipid syndrome (APS) is potentially associated with RPL^4^, ^5^ and is mainly diagnosed using the Sydney classification criteria for APS.^4^ Following APS diagnosis based on these classification criteria, heparin and aspirin (HA) therapy is the primary standard of care.^6^ However, the Sydney classification criteria for APS are stringent and require both clinical and laboratory analyses before a diagnosis is made. Furthermore, laboratory findings must be positive on two or more occasions, at least 12 weeks apart.^4^ Therefore, in several cases, the laboratory findings may be consistent with APS, but the clinical criteria are inconsistent (non‐criteria APS), or the converse is true.^4^, ^7^, ^8^ As the treatment efficacies of low‐dose aspirin (LDA) and HA are similar for non‐criteria APS,^9^, ^10^, ^11^ LDA is generally the preferred treatment approach, particularly for transient APS. Anti‐cardiolipin (anti‐CL) IgG, IgM, anti‐β2‐glycoprotein 1 (anti‐β2GP1) IgG, IgM and lupus anticoagulant (LAC) are the antiphospholipid antibodies included in the Sydney classification criteria. In addition, anti‐phosphatidylethanolamine (anti‐PE) IgG, IgM^10^, ^11^ antibodies and anti‐phosphatidylserine/prothrombin (anti‐PS/PT) antibodies,^12^, ^13^ which are termed as seronegative APS (SNAPS), are also potentially associated with RPL, despite being excluded from the Sydney classification criteria.^14^, ^15^ Treatment with HA or LDA results in fewer miscarriages when these antibodies are positive.^12^, ^16^, ^17^

The European Society of Human Reproduction and Embryology does not recommend screening for hereditary thrombophilia. In Japan, the frequency of protein S is approximately 2%, which is almost 10 times higher than that in the West.^18^ The majority of the protein S deficiency cases with RPL in Japan involve Protein S Tokushima, a genetic mutation in the second epidermal growth factor region unique to the Japanese population.^19^ Hence, it is not appropriate to directly apply global treatment practices in Japan.^19^

The NOHA study has identified factor XII deficiency as a risk factor for miscarriages,^20^ although its acceptance as a risk factor for RPL is still globally controversial.^21^ Sato et al. reported the presence of autoantibodies against factor XII in patients with factor XII deficiency.^22^ In the Japan Agency for Medical Research and Development (AMED) study, protein S and factor XII deficiencies were included in the primary screening, and significantly increased live birth rates were reported with LDA and/or heparin therapy compared to those with no treatment.^23^

Moreover, platelet aggregation is increased in patients with unexplained RPL.^24^, ^25^ Notably, LDA is known to reduce platelet aggregation in other diseases.^26^, ^27^ However, the management of increased platelet aggregation in patients with RPL remains unclear. LDA is commonly administered for thrombophilic predispositions such as antiphospholipid antibodies that do not completely meet the Sydney classification criteria for APS, protein S deficiency, factor XII deficiency and increased platelet aggregation. However, it remains unclear whether LDA therapy should be continued in subsequent pregnancies if it was unsuccessful previously, or if heparin should be administered instead. Therefore, the present study aimed to establish whether it is preferable to repeat LDA or switch to HA when LDA has been previously ineffective in thrombophilic predispositions other than APS and to determine the characteristics of these cases.

## 2 MATERIALS AND METHODS

### 2.1 Study design and participants

This study included 3291 pregnancies of patients with RPL who visited the Department of Obstetrics and Gynecology of Nippon Medical School between 2016 and 2023. RPL was defined as two or more early miscarriages and/or stillbirths. A retrospective cohort study was performed on 913 of 3291 patients who received LDA, based on several clinical parameters.

The inclusion criteria were as follows: (1) laboratory findings not completely consistent with the Sydney classification criteria but consistent with clinical criteria (non‐criteria APS)^7^: (i) positive antiphospholipid antibodies but not fully meeting the criteria for APS‐complicated pregnancy, including transient positivity (one‐time strong positive) of LAC (dRVVT, aPTT), anti‐CLIgG, anti‐CLIgM and anti‐CLβ2GP1 complex antibodies >99th percentile; (ii) weakly positive (anti‐CLIgG, anti‐CLIgM >95th percentile)^28^; or (iii) strongly positive antibodies or two or more instances of antibody positivity at least 12 weeks apart, but not meeting the clinical criteria for obstetric APS (two or one miscarriage); (2) clinical criteria meeting the Sydney Criteria but with transient positivity of SNAPS, such as anti‐PEIgG, anti‐PEIgM or anti‐PS/PT antibodies, defined as one‐time strong positive values for anti‐PEIgG, anti‐PEIgM or anti‐PS/PT value (>99th percentile); (3) protein S deficiency, with protein S levels being <50% of the reference value^29^; (4) factor XII deficiency, with factor XII levels being <50%^30^; and (5) increased platelet aggregation (type 3).^31^ The exclusion criteria included parental chromosome abnormalities or a miscarriage resulting in chromosomal abnormalities.

#### 2.1.1 Determination of thrombophilia

Anti‐CLIgG and IgM antibodies were measured using the MESACUP™ Test (BML Inc., Tokyo, Japan) until March 2022, and the APS panel test was performed after April 2022. Anti‐CLβ2GP1 complex antibodies were tested using the anti‐CLβ2GP1 kit ‘Yamasa’ enzyme immunoassay until March 2022, while the APS panel test was implemented after April 2022.

For the lupus anticoagulant dRVVT and aPTT methods, HemosIL dRVVT (Instrumentation Laboratory Company, Bedford, MA, USA) and HemosIL SCT (HemosIL; Instrumentation Laboratory Company) were used, respectively. Anti‐PE IgG and IgM antibodies were measured using ELISA at SRL Corporation (Tokyo Japan), and anti‐PS/PT antibodies were measured using the PS/PT ELISA Kit (Medical Biological Laboratories, New York, NY, USA). Protein S activity was measured using PS‐Clot (HemosIL; Instrumentation Laboratory Company). Factor XII was measured using Factor XII‐Deficient Plasma Patronin SL (Sysmex Corporation, Kobe, Japan). Platelet aggregation capacity was determined using a turbidimetric method (DS Medical Reagents, Tokyo, Japan).

#### 2.1.2 LDA therapy

Of the 913 patients treated with LDA (aspirin 100 mg once daily), those who had miscarriages due to parental or chorionic chromosome abnormalities were excluded. The remaining patients with clear pregnancy outcomes were divided into the following two groups: (1) live births and (2) miscarriages or stillbirths. The clinical background of the patients was analyzed retrospectively. Cases in which the first LDA treatment resulted in miscarriage and stillbirth were assessed, as well as the outcomes of the subsequent treatments (second, and third or more), with a particular focus on the live birth rate based on the number of LDA attempts.

#### 2.1.3 HA therapy

**FIGURE 1:**
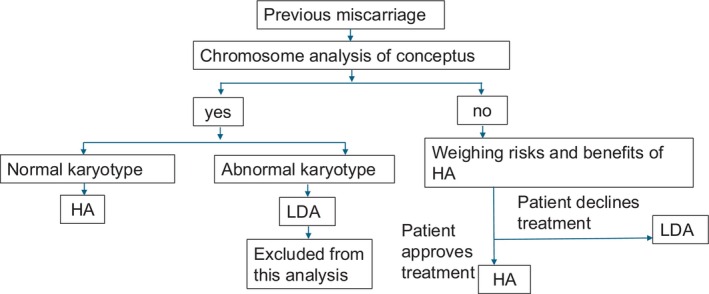
Flow chart of the treatment approach. HA, heparin‐aspirin therapy, LDA, low‐dose aspirin.

A flowchart of the treatment approach is illustrated in Figure 1. The criteria for initiating HA therapy (unfractionated heparin 5000 unit subcutaneously every 12 h, plus aspirin 100 mg) after pregnancy loss due to LDA therapy were as follows: the trophoblastic chromosome analysis of the conceptus confirmed a normal karyotype, or the analysis could not be performed but the patient consented to it. The exclusion criteria were as follows: if the trophoblastic chromosome analysis of the conceptus revealed an abnormal karyotype, or if the patient declined HA therapy and opted for LDA instead.

**FIGURE 2:**
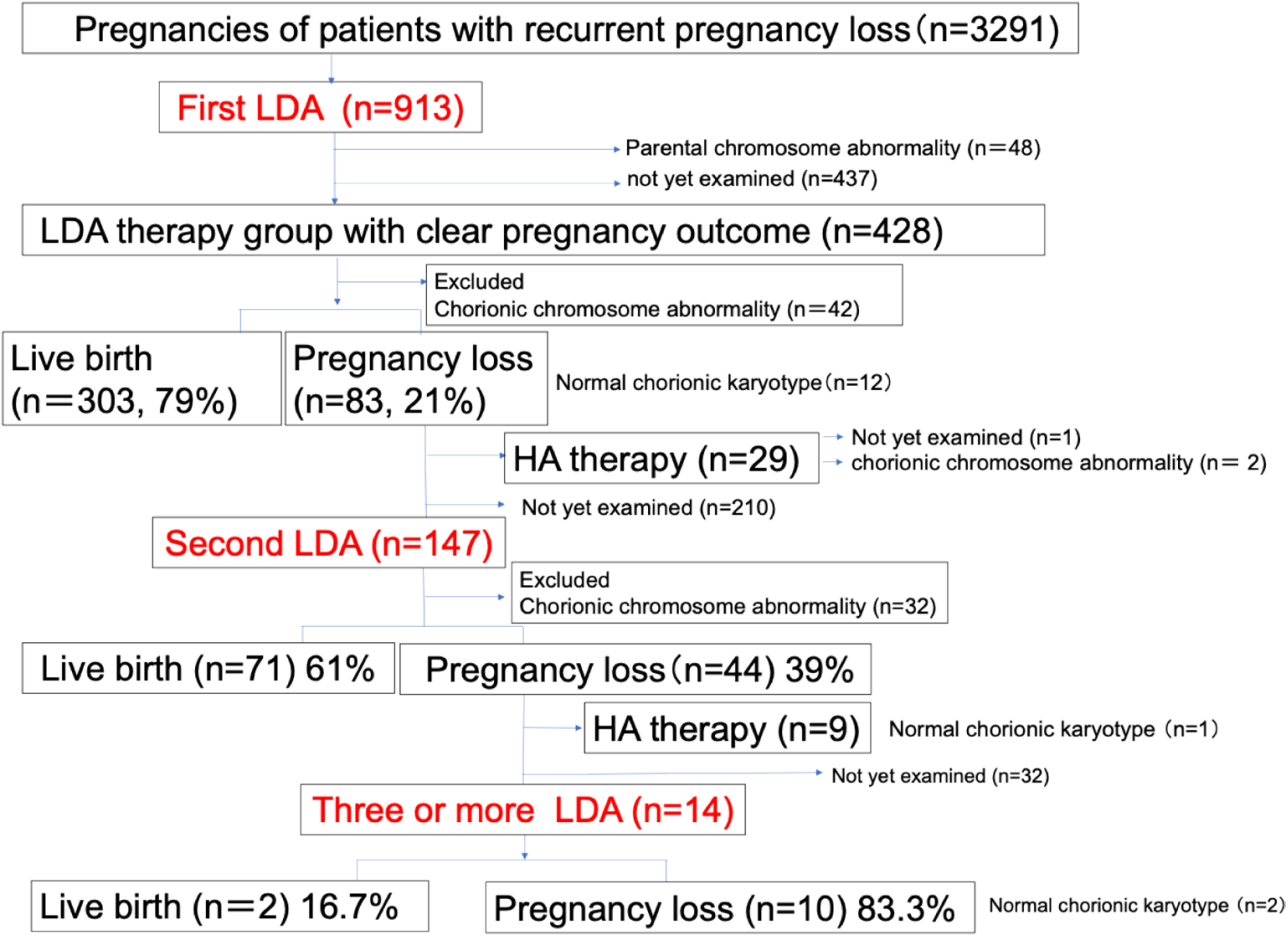
Schematic representation of the study design.

The clinical background and RPL rates of patients who were initially administered LDA but had miscarriages or stillbirths and those who were also treated with LDA for a second time were compared with those treated with HA. Similarly, patients who had undergone ≥2 LDA treatments or HA treatment were retrospectively analyzed (Figure 2).

### 2.2 Ethical approval

The study was approved by the Ethics Committee of Nippon Medical School (B‐2020‐154). All patients were informed of the risk factors, treatment and pregnancy outcomes; written informed consent was obtained from all study participants. This study adhered to the guidelines of the Declaration of Helsinki.

### 2.3 Statistical analyses

The characteristics of thrombophilic predisposition in patients treated with LDA were evaluated in the live birth and miscarriage groups. In addition, in cases of experiencing miscarriage or stillbirth despite LDA therapy, the live birth rate was evaluated and compared between the LDA and HA groups in the next pregnancy. Statistical analyses were performed with Graph Pad Prism 10 Version 10.3.0 (GraphPad Software, San Diego, CA, USA), using the *t*‐test and Fisher's test, and a *p*‐value < 0.05 (two‐tailed) was considered significant.

## 3 RESULTS

### 3.1 Patient characteristics

**FIGURE 3:**
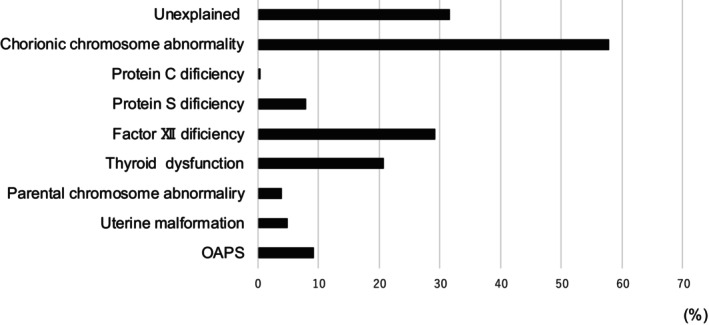
Percentage of causes related to recurrent pregnancy loss. OAPS, obstetrics antiphospholipid syndrome.

Among the 3291 pregnancies with RPL, 41/448 (9.2%) were tested for APS and diagnosed with obstetric APS. Other risk factors for RPL were uterine malformation (160/3291 [4.9%]), parental chromosomal abnormalities (48/1198 [4%]), thyroid dysfunction (469/2254 [20.8%]), factor XII deficiency (677/2311 [29.3%]), protein S deficiency (177/2241 [7.9%]), protein C deficiency (11/2376 [0.05%]) and unexplained causes (1045/3291 [31.7%]). Notably, these factors may overlap (Figure 3). The average number of early miscarriages was 2.28 (range 0–16; *n* = 2906/3264 [89%]), while the average number of late miscarriages after 10 weeks was 0.5 (range 0–2; *n* = 212/2297 [9.2%]). The average number of stillbirths after 22 gestational weeks was 0.0082 (range 0–1; *n* = 35/2325 [0.042%]), and the average parity was 0.37 (range 0–6; *n* = 520/2569 [20.2%]). Preterm births occurred in 3.9% of cases (60/1524), hypertensive disorders in pregnancy in 0.9% (29/3173), and fetal growth restriction in 0.7% (23/3173).

### 3.2 Live birth rate following LDA therapy

The study design is presented in Figure 2. Table 1 depicts the live birth rates of groups with one (78.5%), two (61.0%) and three or more (16.7%) LDA therapy sessions. The live birth rates in groups with three or more LDA treatments were lower than those with one or two LDA treatments.

**TABLE 1:** Live birth rates based on the LDA therapy session.

Frequency of LDA therapy	Live birth rate
First	78.5% (303/386)
Second	61.0% (71/115)
Third and subsequent therapies	16.7% (2/12)

Abbreviation: LDA, low‐dose aspirin.

### 3.3 Clinical backgrounds of patients treated with the first LDA therapy

In terms of age, number of previous miscarriages, or number of previous biochemical pregnancy losses, the clinical backgrounds of patients with live births were not significantly different from those of patients experiencing pregnancy loss following the first LDA therapy (Table 2).

**TABLE 2:** Clinical background of patients treated with the first LDA therapy session.

	Live birth (*n* = 303)	Pregnancy loss (*n* = 83)	*p*‐value
Age (years)	35.4 ± 0.26	35.9 ± 0.50	0.89
Number of previous miscarriages	2.20 ± 0.12	2.46 ± 0.18	0.19
Number of previous chemical pregnancy losses	0.64 ± 0.86	0.87 ± 019	0.16

*Note*: Values are presented as the mean ± standard error of the mean (SEM). The non‐parametric Mann–Whitney test was used to compare the groups.

Abbreviation: LDA, low‐dose aspirin.

### 3.4 Live birth acquisition rates based on thrombophilic predisposition during the first LDA therapy

In the first LDA therapy group, when LAC aPTT was weakly positive, transiently positive or not clinically confirmed as obstetric APS, the birth rates were significantly low, despite treatment with LDA (Live birth: 1.7% [1/60] vs. Pregnancy loss: 12.5% [2/16], birth rates: 33.3%, *p* = 0.048). LAC dRVVT, anti‐CLIgG, anti‐CLIgM and anti‐PEIgG exhibited slightly higher rates of miscarriages; however, this was not statistically significant. For other thrombogenic factors, such as non‐criteria APS with anti‐CLβ2GP1 complex, anti‐PEIgM, anti‐PS/PT antibodies, protein S deficiency, factor XII deficiency and increased platelet aggregation, the first LDA treatment did not improve the live birth rates (Table 3).

**TABLE 3:** Live birth rates based on thrombophilic predisposition during the first LDA therapy.

	Live birth (*n* = 303)	Pregnancy loss (*n* = 83)	Live birth rate	*p*‐value
Anti‐CLIgG (%) (*n*)	4.2 (12/287)	7.7 (6/78)	66.7 (12/18)	0.20
Anti‐CLIgM (%) (*n*)	3.5 (10/284)	7.7 (6/79)	62.5 (10/16)	0.12
Anti‐CLβ2GP1 complex (%) (*n*)	0.7 (2/274)	0.00 (0/59)	100 (2/2)	1.00
LAC (dRVVT) (%) (*n*)	3.1 (9/291)	5.2 (4/77)	69.2 (9/13)	0.370
LAC (aPTT) (%) (*n*)	1.7 (1/60)	12.5 (2/16)	33.3 (1/3)	0.048*
Anti‐PEIgG (%) (*n*)	8.7 (25/286)	12.8 (10/78)	71.4 (25/35)	0.279
Anti‐PEIgM (%) (*n*)	7.4 (21/282)	6.5 (5/77)	80.8 (21/26)	0.775
Anti‐PS/PT antibody (%) (*n*)	9.3 (10/107)	3.4 (1/29)	90.9 (10/11)	0.302
Protein S deficiency (%) (*n*)	15.8 (45/285)	15.2 (12/79)	78.9 (45/57)	0.897
Factor XII deficiency (%) (*n*)	48.3 (139/288)	40.8 (31/76)	81.8 (139/170)	0.245
Increased platelet aggregation (%) (*n*)	37.4 (108/289)	42.3 (33/78)	76.6 (108/141)	0.426

*Note*: Fisher's exact test was used for comparison between groups.

Abbreviations: anti‐CLIgG, anti‐cardiolipin IgG; anti‐CLIgM, anti‐cardiolipin IgM; anti‐CLβ2GP1 complex, anti‐cardiolipin β2GP1; anti‐PE IgG, anti‐phosphatidylethanolamine IgG; anti‐PE IgM, anti‐phosphatidylethanolamine IgM; anti‐PS/PT IgG, anti‐phosphatidylserine/prothrombin antibody; LAC (aPTT), lupus anticoagulant (activated partial thromboplastin time); LAC (dRVVT), lupus anticoagulant (diluted Russell viper venom time); Lupus anticoaglant (activated partial thromboplastin time); LDA, low‐dose aspirin.

* *p* < 0.05.

### 3.5 Clinical background, live birth rate and characteristics of thrombophilic predisposition of patients who underwent a second LDA therapy after experiencing miscarriage and/or stillbirth post‐first LDA therapy

The live birth rate after the second LDA treatment (*n* = 115) was 61.1% (Table 1). In terms of age, the number of previous miscarriages, or the number of previous biochemical pregnancy losses, the clinical backgrounds of patients with live births (*n* = 71) were not significantly different from those of patients with pregnancy losses (*n* = 44) following the second LDA therapy (Table 4).

**TABLE 4:** Clinical background of patients treated with the second LDA therapy.

	Live birth (*n* = 71)	Pregnancy loss (*n* = 44)	*p*‐value
Age (years)	36.6 ± 0.74	36.7 ± 0.96	0.66
Number of previous miscarriages	3.06 ± 0.22	3.82 ± 0.22	0.38
Number of previous chemical pregnancy losses	0.46 ± 0.18	1.25 ± 077	0.42

*Note*: Values are presented as the mean ± SEM. The non‐parametric Mann–Whitney test was used to compare the groups.

Abbreviation: LDA, low‐dose aspirin.

In cases of non‐criteria APS, SNAPS, protein S deficiency, factor XII deficiency, or increased platelet aggregation, the first attempt of LDA resulted in miscarriage and stillbirth, and the second attempt of LDA was subsequently performed. A non‐significantly low live birth rate was observed in cases with non‐criteria APS for anti‐CLIgG antibodies (live birth rate: 33.3%, *p* = 0.0723; Table 5). Other thrombogenic factors, such as non‐criteria APS positive for anti‐CLIgM, anti‐CLβ2GP1 complex, LAC (dRVVT), LAC (aPTT), transiently positive anti‐PEIgG, anti‐PEIgM, or anti‐PS/PT antibodies, protein S deficiency, factor XII deficiency, and increased platelet aggregation, did not improve the live birth rates (Table 5).

**TABLE 5:** Live birth rates based on thrombophilic predisposition following the second LDA therapy.

Thrombophilic predisposition	Live birth (*n* = 71)	Pregnancy loss (*n* = 44)	Live birth rate	*p*‐value
Anti‐CLIgG (%) (*n*)	4.48 (3/67)	15.38 (6/39)	33.3 (3/9)	0.0723
Anti‐CLIgM (%) (*n*)	4.7 (3/59)	8.8 (3/39)	50 (3/6)	0.1267
Anti‐CLβ2GP1 complex (%) (*n*)	0.00 (0/59)	7.69 (0/35)	0 (0/0)	>0.999
LAC (dRVVT) (%) (*n*)	0.00 (0/19)	6.38 (3/28)	0 (0/3)	0.256
LAC (aPTT) (%) (*n*)	0.00 (0/59)	0.00 (0/35)	0 (0/0)	>0.99
Anti‐PEIgG (%) (*n*)	9.84 (6/61)	5.00 (2/40)	75 (6/8)	0.473
Anti‐PEIgM (%) (*n*)	7.94 (5/63)	7.69 (3/39)	62.5 (5/8)	>0.99
Anti‐PS/PT antibody (%) (*n*)	0.00 (0/17)	8.33 (1/12)	0 (0/1)	0.413
Protein S deficiency (%) (*n*)	4.55 (3/66)	2.56 (3/38)	50 (3/6)	0.666
Factor XII deficiency (%) (*n*)	64.62 (42/61)	35.38 (23/41)	64.6 (42/65)	0.212
Increased platelet aggregation (%) (*n*)	60.00 (45/75)	69.23 (45/65)	50 (45/90)	0.291

*Note*: Fisher's exact test was used for comparison between groups.

Abbreviations: anti‐CLIgG, anti‐cardiolipin IgG; anti‐CLIgM, anti‐cardiolipin IgM; anti‐CLβ2GP1 complex, anti‐cardiolipin β2GP1; anti‐PE IgG, anti‐phosphatidylethanolamine IgG; anti‐PE IgM, anti‐phosphatidylethanolamine IgM; anti‐PS/PT IgG, anti‐phosphatidylserine/prothrombin antibody IgG; LAC (dRVVT), lupus anticoagulant (diluted Russell viper venom time); LAC (aPTT), Lupus anticoagulant (activated partial thromboplastin time); LDA, low‐dose aspirin.

### 3.6 Clinical background and pregnancy outcomes in patients who underwent the third or subsequent LDA therapies after experiencing miscarriage and/or stillbirth post‐second LDA therapy

The live birth rate after the third or more LDA treatment session was 16.7% (2/12) (Table 1). The mean age of patients experiencing miscarriages was 34 years; the average number of miscarriages was 4, and the number of biochemical pregnancies was 1. Three positive cases for transient anti‐CLIgG, one for anti‐PEIgM, two for protein S deficiency, one for factor XII deficiency, two for anti‐PS/PS antibody and two for increased platelet aggregation were noted to exhibit thrombophilic predisposition. Moreover, the mean age of patients who had live births following three or more LDA therapies was 37 years old. The results also indicated 3.5 previous miscarriages and one biochemical pregnancy with weakly positive anti‐CLIgM and increased platelet aggregation (Table 6).

**TABLE 6:** Patient background, thrombophilic predisposition and outcomes of LDA therapy after three or more sessions.

No.	Treatment	Age (years)	Number of previous miscarriages	Chemical pregnancy loss	Thrombophilic predisposition	Pregnancy outcome	Pregnancy complications
1	LDA → LDA → LDA	42	2	1	Protein S deficiency	Miscarriage	
2	LDA → LDA → LDA	37	5	2	Increased platelet aggregation	Miscarriage	
3	LDA → LDA → LDA	35	3	1	Anti‐PS/PT antibody	Miscarriage	
4	LDA → LDA → LDA	30	3	1	Transiently positive anti‐CL IgG	Miscarriage	
5	LDA → LDA → LDA	34	2	3	Weakly positive anti‐CL IgG antibody	Miscarriage	
6	LDA → LDA → LDA	32	3	0	Positive anti‐PEIgM antibody	Miscarriage	
7	LDA → LDA → LDA	38	5	2	Factor XII deficiency	Miscarriage	
8	LDA → LDA → LDA	37	4	1	Transiently positive anti‐CL IgM antibody	Live birth	−
9	LDA → LDA → LDA → LDA	43	3	1	Protein S deficiency	Miscarriage	
10	LDA → LDA → LDA → LDA	38	6	2	Increased platelet aggregation	Live birth	−
11	LDA → LDA → LDA → LDA	36	4	1	Positive anti‐PS/PT antibody	Miscarriage	
12	LDA → LDA → LDA → LDA	32	4	1	Transiently positive anti‐CL IgG antibody	Miscarriage	

*Note*: Nos. 2 and 10, and Nos. 3 and 11 are the same patient.

Abbreviations: anti‐CLIgG, anti‐cardiolipin IgG; anti‐CLIgM, anti‐cardiolipin IgM; anti‐PE IgG, anti‐phosphatidylethanolamine IgG; anti‐PE IgM, anti‐phosphatidylethanolamine IgM; anti‐PS/PT IgG, anti‐phosphatidylserine/prothrombin antibody; LDA, low‐dose aspirin.

### 3.7 Comparison of live birth rates between patients who underwent the second LDA therapy and the first HA therapy after experiencing miscarriage and/or stillbirth post‐first LDA therapy

The live birth rates between the HA and LDA therapy groups after first experiencing miscarriage and/or stillbirth post‐first LDA therapy were not significantly different (LDA: 61.74% [71/115] vs. HA: 70.37% [19/27], *p* = 0.57; Table 8).

### 3.8 Live birth rate and thrombophilic predisposition characteristics of patients who underwent HA therapy after experiencing miscarriage and/or stillbirth post‐first LDA therapy

The mean age of the patients who underwent HA therapy after experiencing miscarriage and/or stillbirth post‐second LDA therapy was 35.06 years. The average number of miscarriages was 3.62, and the average number of biochemical pregnancies was 1.10.

Live birth rates based on thrombophilic predisposition in patients who had miscarriages or stillbirths after initial LDA therapy and were treated with HA during the next pregnancy were determined. For non‐criteria APS, SNAPS, or in the case of protein S deficiency, factor XII deficiency, or increased platelet aggregation, there was no significant difference in the live birth rates for any of the thrombophilic predisposing factors. However, 80% (4/5) of the patients with weakly positive or transiently positive anti‐CLIgG had live births after receiving HA therapy (data not shown).

### 3.9 Clinical background and comparison of live birth rates between patients who underwent the third or subsequent LDA and HA therapy after experiencing miscarriage and/or stillbirth post‐second LDA therapy

The mean age of the patients who underwent HA therapy after experiencing miscarriage and/or stillbirth post‐second LDA therapy was 35.75 years The average number of miscarriages was 5.62, and the average number of biochemical pregnancies was 1.25 (Table 7). Two cases positive for transient anti‐CLIgG, two each for anti‐PEIgG and IgM, one for protein S deficiency, three for factor XII deficiency and two for anti‐PS/PS antibody were recorded per thrombogenic predisposition. One patient had two positive thrombophilic predispositions. The two patients who had miscarriages after HA therapy exhibited increased platelet aggregation and factor XII deficiency; the patient with increased platelet aggregation had a history of 16 miscarriages. The patient with factor XII deficiency was 44 years old, and the possibility of a trophoblastic chromosome abnormality could not be ruled out (Table 7). The live birth rate was significantly higher with HA therapy than that with three or more LDA therapies (LDA 16.7% [2/12] vs. HA 75% [6/8], *p* = 0.00194; Table 8).

**TABLE 7:** Patient background, thrombophilic predisposition and outcome of HA therapy after two or more LDA sessions.

No.	Treatment	Age (years)	Number of previous miscarriages	Chemical pregnancy loss	Thrombophilic predisposition	Pregnancy outcome	Pregnancy complications
1	LDA → LDA → HA	36	6	0	Weakly positive anti‐CL IgG antibody, protein S deficiency, anti‐transiently positive PS/PT antibody	Live birth	—
2	LDA → LDA → HA	30	16	2	Increased platelet aggregation	Miscarriage	
3	LDA → LDA → HA	44	3	2	Factor XII deficiency	Miscarriage	
4	LDA → LDA → HA	36	3	0	Transiently positive Anti‐PEIgM antibody Factor XII deficiency	Live birth	—
5	LDA → LDA → LDA → HA	35	3	3	Transiently positive anti‐CL IgG antibody =	Live birth	—
6	LDA → LDA → LDA → HA	33	4	2	Positive anti‐PEIgM antibody positive	Live birth	26‐week Premature delivery
7	LDA → LDA → LDA → HA	38	5	0	Factor XII deficiency	Live birth	—
8	LDA → LDA → LDA → LDA → HA	36	7	0	Anti‐PS/PT antibody	Live birth	HDP, 36‐week Premature delivery

*Note*: No. 6 in Table 6 and No. 6 in Table 7 are the same patient; Nos. 3, 11 in Table 6 and No. 8 in Table 7 are the same patient.

Abbreviations: anti‐CLIgG, anti‐cardiolipin IgG; anti‐CLIgM, anti‐cardiolipin IgM; anti‐PE IgG, anti‐phosphatidylethanolamine IgG; anti‐PE IgM, anti‐phosphatidylethanolamine IgM; anti‐PS/PT IgG, anti‐phosphatidylserine/prothrombin antibody; HA, heparin‐aspirin therapy; LDA, low‐dose aspirin.

**TABLE 8:** Comparison of the live birth rate for LDA and HA therapies.

Frequency of LDA therapy	Live birth rate for LDA therapy	Frequency of HA therapy	Live birth rate for HA therapy	*p*‐value
First	78.5% (303/386)			
Second	61.74% (71/115)	HA therapy after 1st LDA therapy	70.37% (19/27)	0.57
Third and subsequent therapies	16.7% (2/12)	HA therapy after 2nd LDA therapy	75.0% (6/8)	0.019*

*Note*: Fisher's exact test was used for comparison between groups.

Abbreviations: HA, heparin‐aspirin therapy; LDA, low‐dose aspirin.

* *p* < 0.05.

## 4 DISCUSSION

The present study suggests that HA therapy should be recommended to increase the rate of live births in patients with thrombophilia who do not meet the diagnostic criteria for APS – non‐criteria APS, SNAPS, protein S deficiency, factor XII deficiency or increased platelet aggregation – if no live births occurred after two or more LDA treatments. Our results revealed a decreased live birth rate despite treatment with LDA in cases of non‐criteria APS with positive LAC aPTT, indicating that the treatment was ineffective. Although the efficacies of LDA and HA were comparable for thrombophilia other than APS, if repeated miscarriages occur after LDA administration, LDA is considered ineffective, and HA therapy should be suggested.

The effectiveness of LDA for thrombophilic predisposition to RPL remains controversial. No efficacy or non‐consistent effects of LDA have been reported.^32^ However, for transient antiphospholipid antibodies (aPL), the live birth rates for LDA and heparin therapy are similar.^23^ The European Alliance of Associations for Rheumatology (EULAR) recommends LDA for asymptomatic aPL with obstetric APS.^33^ In a previous meta‐analysis, LDA was found to decrease obstetric complications for asymptomatic aPL (OR:0.25 95% Cl: 0.10–0.62).^34^ For factor XII and protein S deficiency, LDA treatment results in a significantly higher live birth rate than that with no treatment, comparable to that with heparin treatment.^23^ Therefore, for thrombophilic predispositions, such as non‐criteria APS and SNAPS, LDA may be the preferred first‐line treatment, which had a response rate of 78.5% in the present study. The EAGeR study indicated that LDA improves the live birth rate in patients with a history of miscarriages at ≤20 weeks of gestation within 1 year, but not in those with one or two miscarriages, regardless of timing.^35^ Therefore, our study may have included patients who did not require LDA at the time of initial administration.

LAC is more strongly associated with thrombosis than anti‐CLIgG is, and the disease is proportional to the antibody levels.^36^ LAC has been reported to be associated with pre‐eclampsia (OR 2.34; 95% CI:1.18–4.64) and fetal growth restriction (OR 4.65; 95% CI: 1.29–16.71); late fetal loss (OR 4.73; 95% CI: 1.08–20.81) has been reported.^37^, ^38^ Given that LAC is a significant factor influencing RPL, it is necessary to consider the patient's history, such as the number of previous miscarriages, when determining the treatment plan.

Clinically, APS is suspected; however, approximately 40% of patients report at least one positive aPL test, even if the aPL findings are not persistently positive.^39^ In particular, the European Registry on Obstetrics Antiphospholipid Antibody Syndrome project study found that the fetal –maternal outcomes are similar in treating the obstetric APS group and the group not classified as APS or the group that non‐clinically fulfils the requirements for APS (non‐criteria obstetric APS: NC‐OAPS).^35^, ^40^ Regarding NC‐OAPS, LDA is considered the standard therapy, as no significant difference in live birth rates has been reported between LDA and heparin therapies.^35^, ^41^ However, in the present study, the rate of live births improved significantly with heparin therapy compared to LDA therapy when repeated LDA treatment failed to result in a live birth. Therefore, heparin therapy may be considered when aspirin therapy is unsuccessful in non‐criteria APS cases.

In the present study, OAPS was diagnosed using the Sydney Criteria classification. However, new classification criteria for APS have recently been provided by ACR/EULAR.^31^ Based on this classification, clinical and laboratory criteria are scored to make the final diagnosis of APS. However, even if the clinical criteria (three or more early miscarriages) and laboratory criteria are met, the score may still not be sufficient for a diagnosis of APS. In such cases, obstetric hypertensive disorders or placental insufficiency before 34 weeks of gestation must also be present to make the diagnosis of APS. Therefore, if the ACR/EULAR classification approach is adopted, heparin therapy would not be recommended for patients with repeated early miscarriages. However, this classification system is not supported by evidence that heparin therapy is effective for repeated early miscarriages in OAPS,^6^ making it difficult to adopt from an obstetric perspective. Therefore, it would be more appropriate to continue following the Sydney Criteria classification.

The benefits of heparin include its role as an anticoagulant therapy. Additionally, heparin may prevent miscarriages through mechanisms such as complement inhibition^42^ and preventing β2gp1 from attaching to antibodies.^43^ Heparin may also prevent miscarriages through effects beyond anticoagulation.^44^ For example, its anti‐inflammatory effects reduce miscarriages in patients with APS in RPL.^44^ In vitro, heparin downregulates inflammatory cytokines and NFκB in human monocytes activated by lipopolysaccharides (LPS).^45^ In a mouse model of sepsis, heparin protects the macrophage glycocalyx by inhibiting heparanase, in addition to its anticoagulant effect, and induces an anti‐inflammatory effect by inhibiting caspase‐11 activation by LPS.^46^ Therefore, the mechanism underlying non‐criteria APS in this study may be attributed to these inflammatory effects rather than thrombogenesis.

To the best of our knowledge, this is the first study to present a strategy for cases of miscarriage despite LDA therapy. However, the study had some limitations. The hereditary thrombogenic predispositions, protein C deficiency, antithrombin deficiency and factor V Leiden mutation were not considered, given their low prevalence in Japan. The efficacy of LDA may vary with acquired and hereditary thrombophilic predisposition; however, these parameters were uniformly evaluated. Furthermore, this is a single‐center, retrospective study that did not include comprehensive parental chromosome testing or chorionic villi in the miscarriage and stillbirth cases. Moreover, in other countries, heparin is typically administered as a low‐molecular‐weight compound, which differs from the unfractionated heparin used in Japan and may have different requirements. Finally, logistic regression analysis was performed on birth rates based on thrombogenic predisposition treated with LDA. However, the results could not be obtained owing to many missing values, as LAC, aPTT and anti‐PS/PT measurements were introduced midway through the study. In the future, we plan to conduct a more comprehensive analysis using the data collected from these tests.

In summary, for thrombophilic predispositions, such as non‐criteria APS, SNAPS, protein S deficiency, factor XII deficiency and increased platelet aggregation, the live birth rate increases with the administration of HA rather than repeated LDAs. The continuation of LDA therapy in subsequent pregnancies, despite prior LDA administration, is not effective. In the future, we intend to expand the indication of heparin by accumulating more cases and clarifying its efficacy for thrombophilic predispositions other than APS.

## CONFLICT OF INTEREST STATEMENT

The authors declare no conflict of interest.

## ETHICS STATEMENT

The Committee of Nippon Medical School Hospital approved the collection and use of biological materials for this study, and all experiments were performed according to the relevant guidelines (19‐03‐56).

## HUMAN RIGHTS STATEMENTS AND INFORMED CONSENT

All experiments were performed in accordance with the ethical standards of the responsible committee on human experimentation and the Helsinki Declaration of 1964 and its later amendments. In this study, human blood samples were analyzed, and written informed consent was obtained from all participants.
